# Transcriptomic analyses of regenerating adult feathers in chicken

**DOI:** 10.1186/s12864-015-1966-6

**Published:** 2015-10-06

**Authors:** Chen Siang Ng, Chih-Kuan Chen, Wen-Lang Fan, Ping Wu, Siao-Man Wu, Jiun-Jie Chen, Yu-Ting Lai, Chi-Tang Mao, Mei-Yeh Jade Lu, Di-Rong Chen, Ze-Shiang Lin, Kai-Jung Yang, Yuan-An Sha, Tsung-Che Tu, Chih-Feng Chen, Cheng-Ming Chuong, Wen-Hsiung Li

**Affiliations:** Biodiversity Research Center, Academia Sinica, Taipei, 11529 Taiwan; Institute of Ecology and Evolutionary Biology, National Taiwan University, Taipei, 10617 Taiwan; Whole-Genome Research Core Laboratory of Human Diseases, Chang Gung Memorial Hospital, Keelung, 20401 Taiwan; Department of Pathology, Keck School of Medicine, University of Southern California, Los Angeles, CA 90033 USA; Department of Animal Science, National Chung Hsing University, Taichung, 40227 Taiwan; Center for the Integrative and Evolutionary Galliformes Genomics (iEGG Center), National Chung Hsing University, Taichung, 40227 Taiwan; Integrative Stem Cell Center, China Medical University, Taichung, 40402 Taiwan; Department of Ecology and Evolution, University of Chicago, Chicago, IL 60637 USA

**Keywords:** Feather, Chicken, Development, Transcriptome, RNA-seq

## Abstract

**Background:**

Feathers have diverse forms with hierarchical branching patterns and are an excellent model for studying the development and evolution of morphological traits. The complex structure of feathers allows for various types of morphological changes to occur. The genetic basis of the structural differences between different parts of a feather and between different types of feather is a fundamental question in the study of feather diversity, yet there is only limited relevant information for gene expression during feather development.

**Results:**

We conducted transcriptomic analysis of five zones of feather morphologies from two feather types at different times during their regeneration after plucking. The expression profiles of genes associated with the development of feather structure were examined. We compared the gene expression patterns in different types of feathers and different portions of a feather and identified morphotype-specific gene expression patterns. Many candidate genes were identified for growth control, morphogenesis, or the differentiation of specific structures of different feather types.

**Conclusion:**

This study laid the ground work for studying the evolutionary origin and diversification of feathers as abundant data were produced for the study of feather morphogenesis. It significantly increased our understanding of the complex molecular and cellular events in feather development processes and provided a foundation for future studies on the development of other skin appendages.

**Electronic supplementary material:**

The online version of this article (doi:10.1186/s12864-015-1966-6) contains supplementary material, which is available to authorized users.

## Background

The genetic and developmental basis of morphological complexity is one of the most important issues in evolutionary biology [1, 2]. Avian feather provides an excellent system for studying the evolution and development of novel morphological traits because it has diverse forms [3–6], and the complex structure of feathers allows for various types of morphological changes to occur. Yet, feathers are homologous with the simpler scales of reptiles and could have evolved from a scale-like epidermal appendage of dinosaurian ancestors of birds [7–10].

Feathers have evolved to have different forms in color, morphology and mechanical properties not only among different bird species, but also among different body regions of a bird individual, giving us an excellent model to study the molecular basis of phenotypic variation of an important structure in a single species. The feather has been used as a model to study morphogenesis of skin appendages [11, 12]. Several candidate genes have been found to be involved in feather formation [13]. For examples, barb and rachis are formed by a periodic invagination and regulated by BMP, NOG, SPRY, and FGF. Moreover, the basal branch pattern is formed by differential cell death and regulated by NCAM, SHH, and caspase. In addition, radial, bilateral symmetric, and asymmetric branching patterns are formed by modulating basal branching circuit that is involved the WNT3A gradient and SPRY. However, the genetic basis of feather variation is still largely unknown. A better understanding of the molecular dynamics associated with the process of feather growth will provide insight into the evolution of diverse feather structures.

The feather is also an excellent example of exaptation. Feathers initially might have evolved for heat regulation, but were then co-opted for display, and later co-opted for flight. These and other evolutionary novelties probably have occurred by changing the expression patterns of genes involved in feather development. The evolutionary co-option of plesiomorphic molecular signaling modules allows for the morphological innovations of feathers to originate and evolve [14, 15].

A hypothesis of morphological evolution postulates that form evolves largely through altering the expression of conserved genes [2]. The molecular and developmental mechanisms that produce the diversification of feather are still poorly understood. The epithelium and the mesenchyme are two major components in feather follicles [16–18]. The epithelium includes both the epithelium enwrapping the mesenchyme and the feather wall epithelium that is connected with the interfollicular epidermis. The mesenchyme includes the dermal papilla and the pulp [19, 20]. The invagination of the multilayered epithelium in the ramogenic zone starts branching morphogenesis. The rachis is formed by fusion of barb ridges at the anterior end of the feather. The marginal plate in basal layer flanking each barb ridge and axial cells undergo apoptosis after the barbule plates are keratinized. The feather branches open in the more mature distal end after the apoptosis of feather sheath and pulp epithelium. Thanks to the feasibility of experimental manipulation and observation, feather regeneration can be analyzed in a comprehensive way and has been proposed to be a unique model for understanding organogenesis [11].

High-throughput sequencing technologies have been applied to characterize transcriptome architectures [21–26]. Systems biology study provides a new technology platform that can reveal molecular expression profiles associated with different morphological developments. Bioinformatic analyses are used to identify genes associated with feather and scale differences [27]. These technologies and skills were used in this study.

**Fig. 1:**
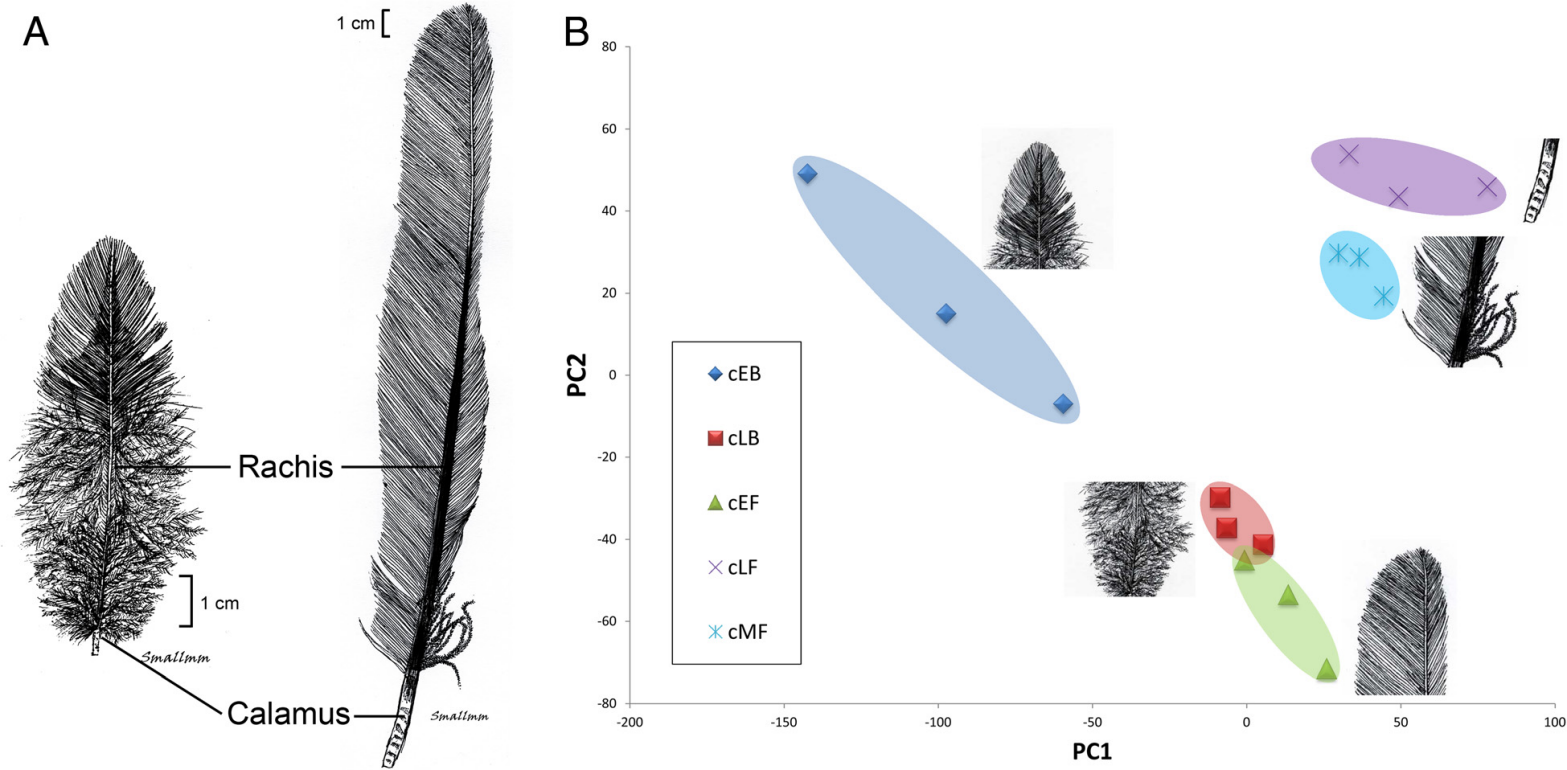
Principal component analysis of gene expression profiles. (**a**) Morphology of body (left) and flight feathers (right). (**b**) Principal Component Analysis (PCA) of gene expression profile. The results were obtained by analyzing 12,608 genes with FPKM >0.1 in all libraries. cEB, early body feather of chicken (pennaceous); cLB, late body feather (plumulaceous); cEF, early primary flight feather; cMF, middle primary flight feather; cLF, late primary flight feather (calamus)

The main goal of this study was to identify differentially expressed genes between different portions of feather using RNA-Seq. We characterized and quantified mRNAs that are expressed in the feather base during feather development in the domestic chicken. Feathers develop from the distal end to the proximal end in a temporal-spatial manner, thus providing an opportunity to analyze gene expression profiles associated with different zones of a feather (Fig. 1a) [5]. Two zones of body feather and three zones of flight feather were selected to represent morphological, structural, and mechanical property differences in feathers (Additional file 1: Figure S1). We made four comparisons: 1) between pennaceous and plumulaceous portions of body feather for understanding how the morphological differences between two parts of a body feather arise; 2) between the pennaceous portions of body feather and flight feather for understanding differences in physical and mechanical properties; 3) between the distal pennaceous portion and the proximal pennaceous portion of flight feather for understanding how the morphological differences between two parts of a flight feather arise; 4) between the proximal pennaceous portion and the calamus of a flight feather for understanding how the barb and rachis are differentiated. These analyses shed light on the genetic basis of feather diversity.

## Results and discussions

### Transcriptome data

We used Illumina Hiseq 2000 to obtain five feather transcriptomes from the pennaceous and plumulaceous portions of body feather, the distal and proximal parts of flight feather, and the flight feather calamus (Additional file 1: Figure S1). Each sample was a pool of mRNA from two-three follicle epidermis of one individual. Three cDNA libraries with insert lengths ranging from 300 to 400 bp were constructed for each sample. The RNA-seq data had been used previously to study the expression pattern of α-and β-keratin genes [28]. In this study we conducted a detailed analysis of the expression patterns for all genes (Additional file 2: Table S1).

To validate the expression data obtained by RNA-Seq, ten genes were selected from the differentially expressed protein-coding genes to perform real time quantitative PCR (RT-qPCR) assays. Reproducibility of the data was confirmed by the strong correlation between the values of gene expression obtained by RNA-Seq and RT-qPCR (Additional file 1: Figure S2). When the pattern of gene expression levels was compared, strong correlations (*R*^2^) ranging from 0.833 to 0.998 between RT-qPCR and RNA-Seq platforms were observed for 90 % of the expressed genes exception for one sample (with *R*^2^ = 0.618), confirming the high reproducibility of the data.

**Fig. 2:**
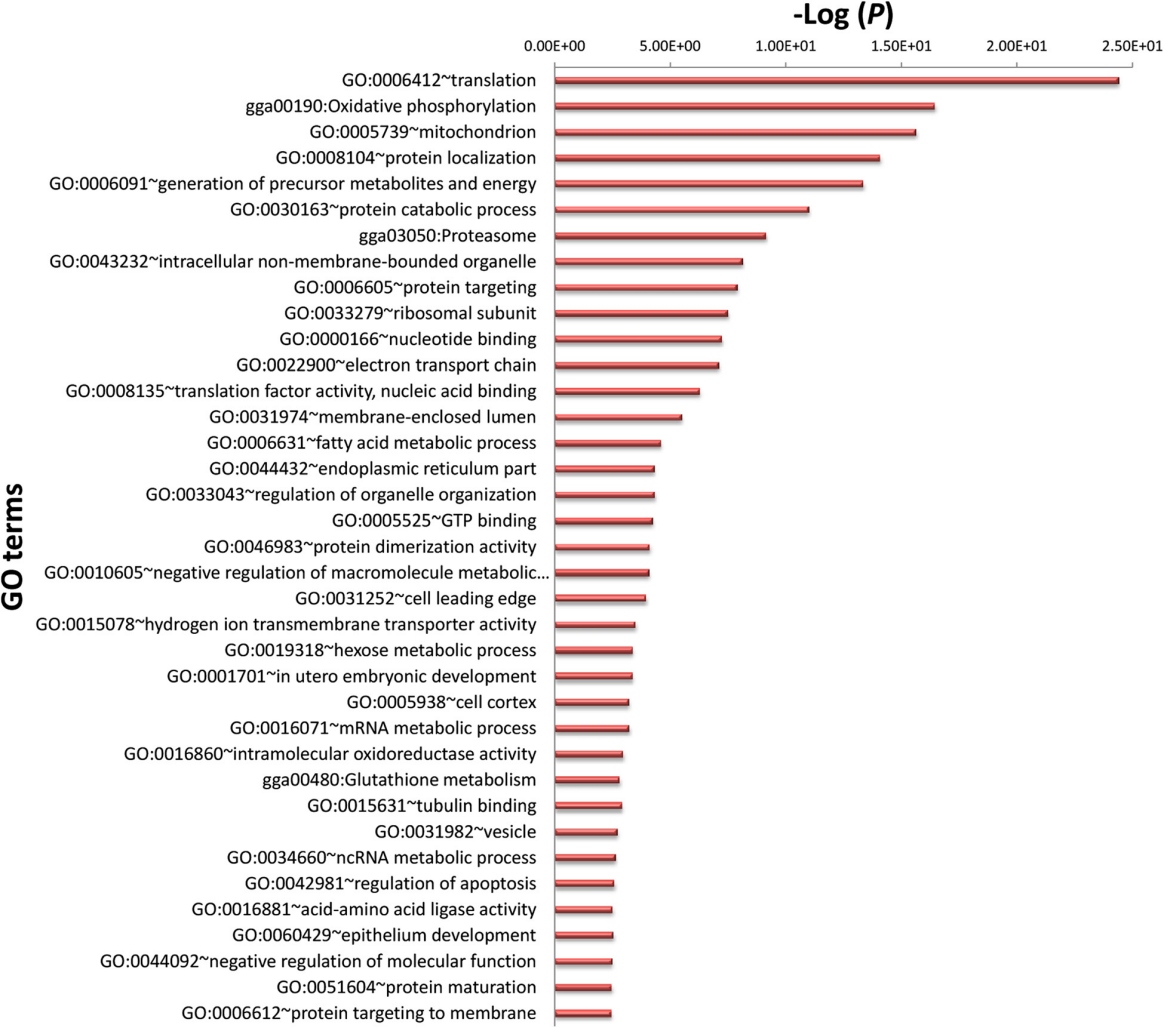
Gene ontology (GO) annotation for top 3,000 transcripts that were highly expressed in feather epithelium

For those genes with a FPKM > 0.1 (FPKM = Fragments Per Kilobase of transcript per Million mapped reads), there were 12,608 genes expressed in all three biological replicates of at least one type of the feather epithelium in the total transcriptomes. Principal components analysis (PCA) showed that samples from the same group clustered together (Fig. 1b). This observation suggests that each sample harbors transcriptomic features that are unique to the feather types or regions. The genes identified in the GO enrichment analysis of the most abundant transcripts in these samples are involved in protein translation, reflecting the rapid production of a protein-made structure (Fig. 2). These results are consistent with the efficient biosynthesis of proteins in the feather follicles. Genes involved in developmental morphogenesis as well as cytoskeletons are also highly abundant in developing feather epithelia.

**Fig. 3:**
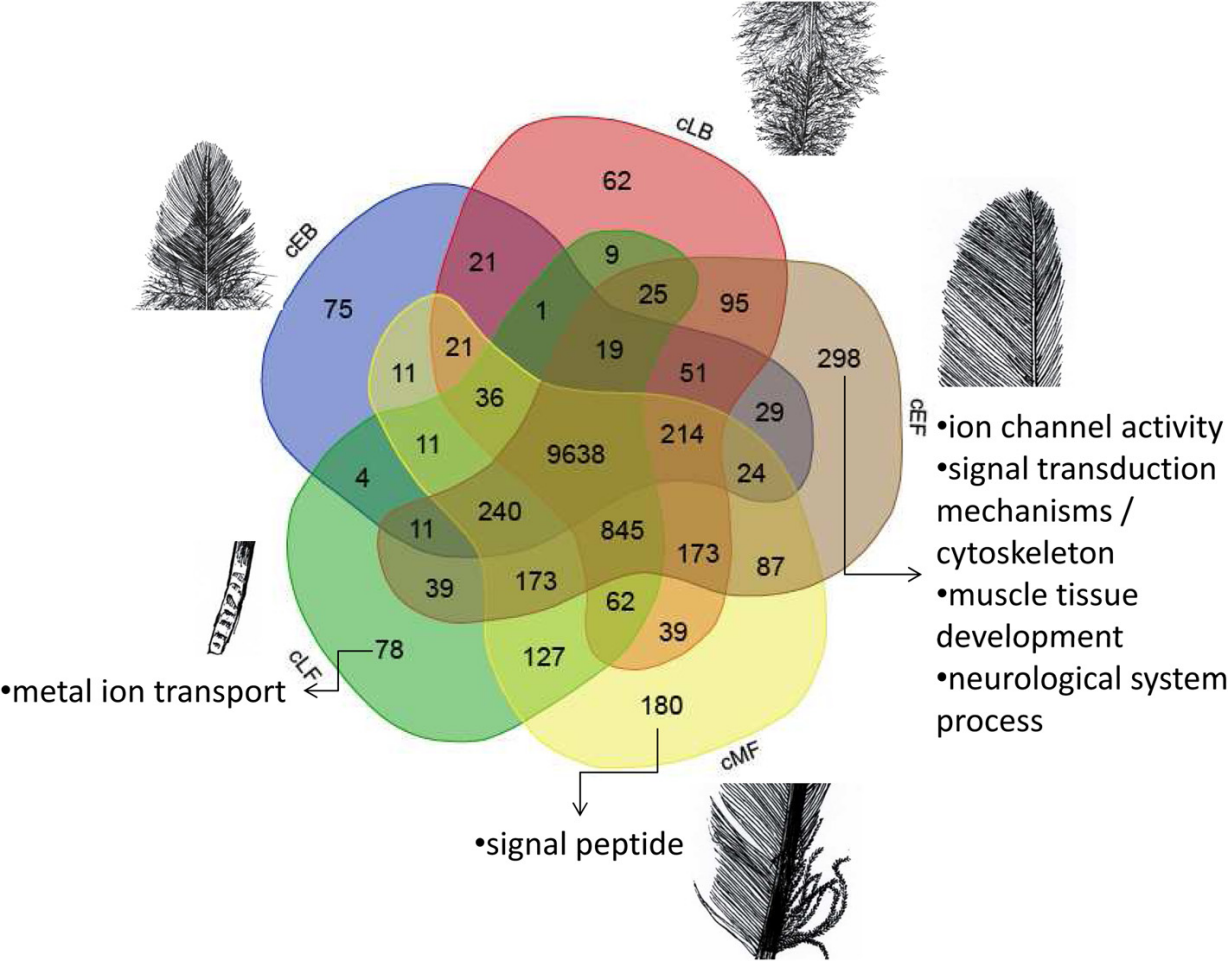
Venn diagram showing the genes expressed in each of the five feather tissue types. Among these genes, 9,638 are expressed at all five samples, 10,001 are co-expressed in cEB and cLB, 10,173 are co-expressed in cEB and cEF, 11,283 are co-expressed in cEF and cMF, and 11,132 are co-expressed in cMF and cLF. The GO enrichment analysis showed that the specifically expressed genes of the body feather (cEB and cLB) are not significantly enriched for any known functions, whereas those of the flight feather (cEF, cMF, and cLF) are significantly enriched for several functions indicated in the figure

An aggregate total of 13,973 expressed genes was expressed (FPKM > 0.1) in at least one of the 15 samples (Additional file 4: Table S3), among which 9,638 genes were expressed in all 15 samples (Fig. 3). In general, the flight feather has more specifically expressed genes than the body feather. The GO enrichment analysis showed that the specifically expressed genes of the body feather are not significantly enriched for any known functions, whereas those of the flight feather are significantly enriched for several functions (Fig. 3). We also found that 49 genes previously identified to have undergone rapid evolution and/or positive selection in avian lineages [29] are expressed in all feather samples (Additional file 5: Table S4). Most of these rapidly evolving and/or positively selected genes are enriched for cytoskeleton and cell adhesion. These proteins may have evolved new functions or properties in feathers and it is worth further investigation.

### Transcriptomic comparison between distal and proximal body feathers

**Fig. 4:**
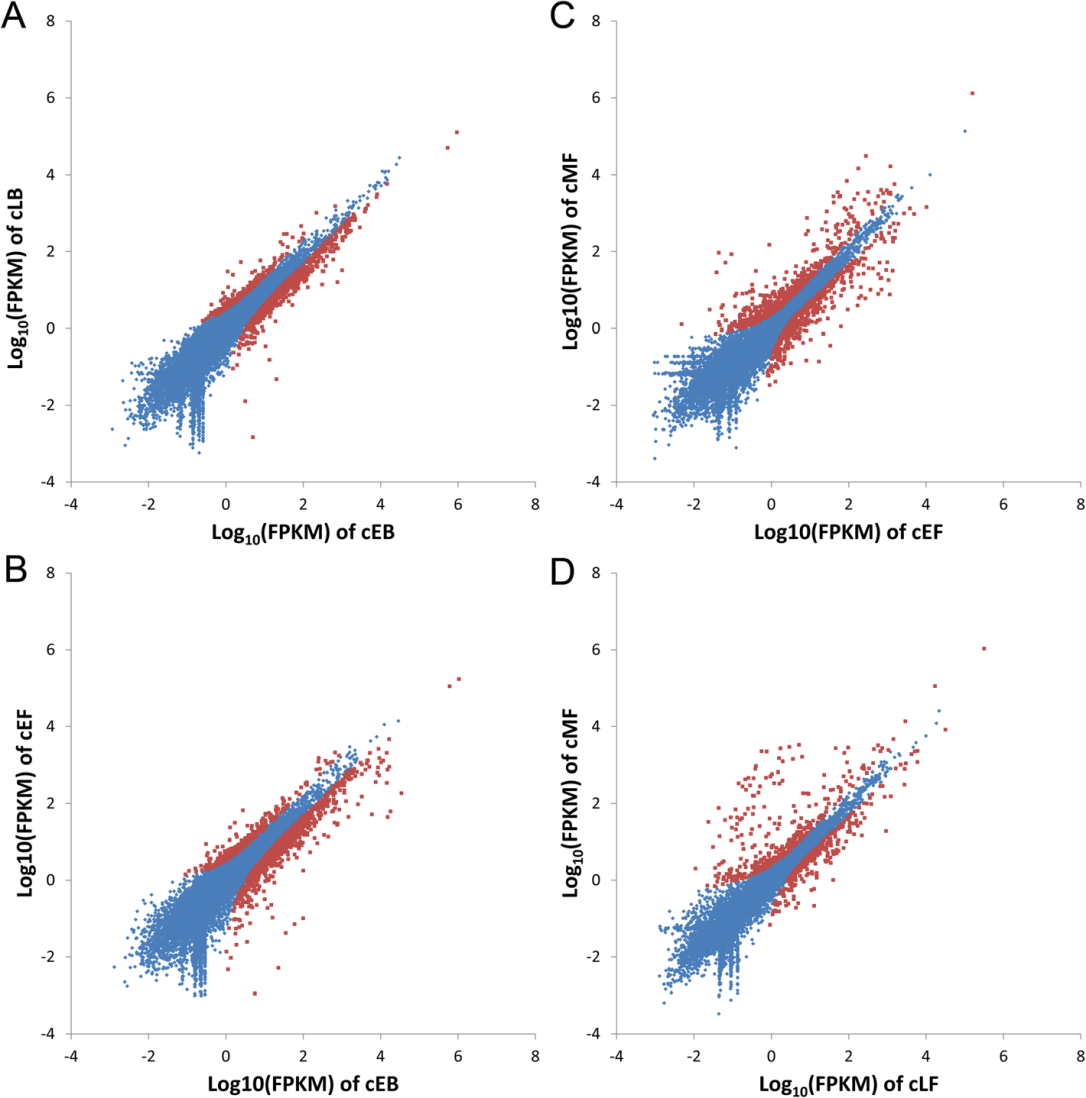
Gene expression level in five comparisons. X-axis and Y-axis plots gene expression counts after FPKM quantification in comparison. (**a**) cEB vs. cLB, (**b**) cEB vs. cEF, (**c)** cEF vs. cMF, and (**d**) cMF vs. cLF. The red points indicate significantly differentially expressed genes

**Fig. 5:**
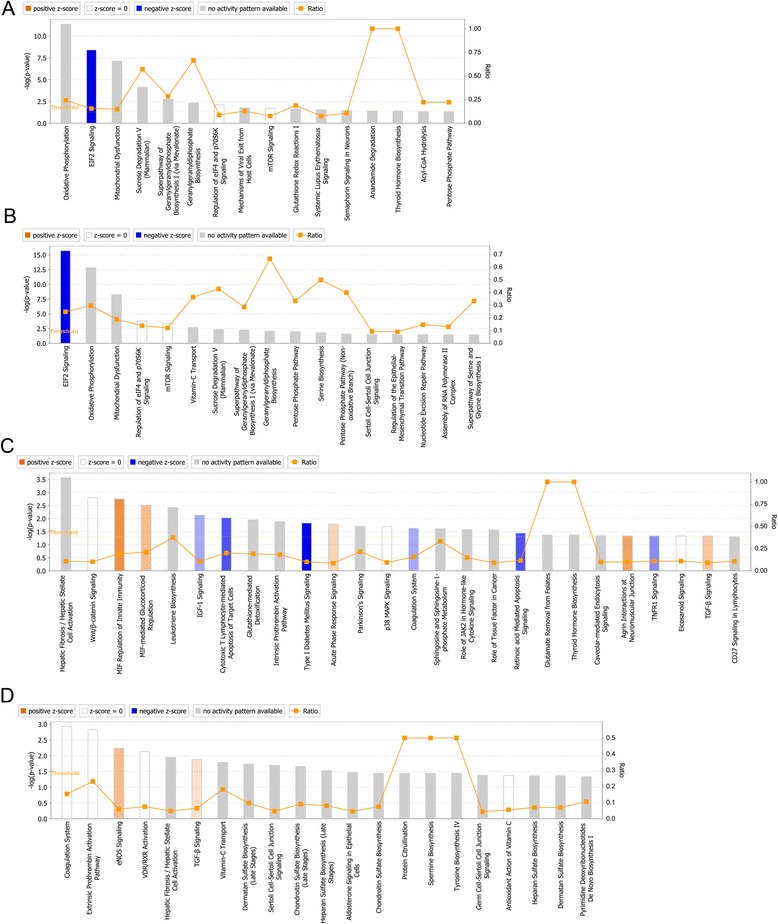
IPA Canonical Pathway analysis of differentially expressed genes. (**a**) cEB vs. cLB, (**b**) cEB vs. cEF, (**c**) cEF vs. cMF, and (**d**) cMF vs. cLF

The distal end of a feather forms earlier than the proximal end and the structure and shape of a body feather change dynamically from the distal end to the proximal end. The distal end of a body feather is mainly pennaceous while the barbs of the proximal end become plumulaceous (Additional file 1: Figure S1). Among the 957 differentially expressed protein-coding genes (DEGs), 223 were up-regulated, while 734 were down-regulated in the plumulaceous portion in comparison to the pennaceous portion (Fig. 4a, Additional file 6: Table S5). IPA (Ingenuity Pathway Analysis) canonical pathway analysis showed that several genes involved in semaphorin signaling in neurons (*PLXNA1*, *NRP1*, *DPYSL3*, *MAPK1*, *CDK5*) were differentially expressed between pennaceous and plumulaceous body feathers (Fig. 5, Additional file 10: Table S9). Semaphorin signaling is known to play an important role in intersomitic vessels, lung, and kidney branching morphogenesis [30, 31], but has not been reported to play any role in feather morphogenesis. A bone morphogenetic protein, BMP2, was predicted to be the upstream regulator for gene expression differences (Table 1). Level of BMP activity has been shown to determine barb ridge branching morphogenesis [19]. Interactions between activators and inhibitors involving Sonic hedgehog (SHH) and BMP2 have been suggested to be involved in the formation of barb ridges in feathers [32].

**Table 1:** Predicted upstream regulators from IPA

Comparison	Upstream regulator	Molecule type	*p*-value of overlap
A	BMP2	growth factor	4.00E-02
B	E2F1	transcription regulator	2.95E-03
	let-7	microRNA	2.72E-02
	RB1	transcription regulator	2.72E-02
C	SP1	transcription regulator	1.15E-03
	CALR	transcription regulator	1.75E-03
	HGF	growth factor	1.75E-03
	RELA	transcription regulator	4.67E-03
	ELF3	transcription regulator	5.10E-03
	CREB1	transcription regulator	5.10E-03
	PPARG	ligand-dependent nuclear receptor	9.92E-03
	PRKCA	kinase	9.92E-03
	TGFB1	growth factor	1.45E-02
	let-7	microRNA	1.61E-02
	NFKB1	transcription regulator	1.61E-02
	ACTG1	other	1.61E-02
	HNRNPA2B1	other	3.04E-02
	HDAC6	transcription regulator	3.20E-02
	CD9	other	4.14E-02
	NCOR1	transcription regulator	4.19E-02
	TRIM16	transcription regulator	4.19E-02
	GLI1	transcription regulator	4.19E-02
	PRKCI	kinase	4.19E-02
	EED	transcription regulator	4.19E-02
	CD44	enzyme	4.19E-02
	RFXAP	transcription regulator	4.19E-02
	PTGER4	g-protein coupled receptor	4.19E-02
	HAS2	enzyme	4.19E-02
	DUSP1	phosphatase	4.19E-02
	MECP2	transcription regulator	4.19E-02
	DNMT3A	enzyme	4.19E-02
	SPHK1	kinase	4.19E-02
	CTSB	peptidase	4.19E-02
	TP63	transcription regulator	4.19E-02
	TUBB3	other	4.19E-02
	EZH2	transcription regulator	4.19E-02
	MMP2	peptidase	4.19E-02
	JAK2	kinase	4.19E-02
	F2	peptidase	4.19E-02
	SIN3A	transcription regulator	4.19E-02
	BAG3	other	4.19E-02
	PRKD1	kinase	4.19E-02
	PRC1	other	4.19E-02
	RCOR1	transcription regulator	4.19E-02
	RFX5	transcription regulator	4.19E-02
	IL1B	cytokine	4.29E-02
D	let-7	microrna	3.06E-03
	F2	peptidase	1.78E-02
	SP1	transcription regulator	1.86E-02
	HNRNPA2B1	other	2.13E-02
	NR3C1	ligand-dependent nuclear receptor	3.20E-02
	ZNF148	transcription regulator	3.54E-02
	NEUROD1	transcription regulator	3.54E-02
	CALR	transcription regulator	3.54E-02
	EGR1	transcription regulator	3.54E-02
	MAPK7	kinase	3.54E-02
	ATF2	transcription regulator	3.54E-02
	IFNG	cytokine	4.88E-02

Among the up-regulated genes in the pennaceous portion compared to the plumulaceous portion of the body feather, the expression of genes involved in protein translation, oxidative phosphorylation, pyrimidine metabolism, ribosomal subunit, contractile fiber part, Peptidyl-prolyl cis-trans isomerase activity, inorganic cation transmembrane transporter activity was increased significantly (Table 2). The up-regulation of genes in these groups may be due to the need of large quantities of protein synthesis in the pennaceous portion of the body feather.

**Table 2:** Functional enrichment analysis of the DEGs in different transcriptomes by the DAVID functional annotation clustering tool

Comparison	Tissue	Up-regulated	Representative annotation terms	Enrichment score	Genes
A	cEB vs. cLB	cEB	Translation	11.57	*COG8, RPL22, HARS, RPL19, RPL10A, YARS, DENR, RPS14, RPS4, MRPL16, RPL13, MRPL23, RPL30, MRPL2, RPL18A, RPS8, RPL37A, RPSA, MRPL24, RPS15, EEF1D, RPLP1, RPL8, RPS7, RPL31, RPL17, MRPS12, RPLP0, RPS28*
			Oxidative phosphorylation	2.99	*NDUFA2, UQCR11, NDUFA8, ATP5A1W, NDUFB6, NDUFS5, PPA1, COX4I1, NDUFAB1, UQCRQ, COX6A1, COX15, ATP5H, UQCR10, NDUFB3, NDUFA1, NDUFS6, COX8A, COX5A, ATP5O, UQCRH, COX6C, NDUFA11*
			Hydrogen ion transmembrane transporter activity	2.78	*ATP5A1W, ATP5O, COX4I1, COX6C, COX5A, LOC770937, UQCRH, UQCRQ*
			Pyrimidine metabolism	1.61	*NME2, ITPA, POLR2L, POLR2H, POLR1C, CANT1, POLR3H, RPB6*
			Contractile fiber part	1.37	*MYL4, TNNC1, HSPB1, CRYAB*
			Ribosomal subunit	1.24	*RPL19, RPS14, RPSA, RPS15, RPL17, MRPS12*
			Peptidyl-prolyl cis-trans isomerase activity	1.22	*PPIB, FKBP25, FKBP1B*
			Inorganic cation transmembrane transporter activity	1.10	*ATP5A1W, COX4I1, UQCRQ, UQCR10, RHBG, COX5A, ATP5O, UQCRH, COX6C*
		cLB	Amino acid transmembrane transporter activity	2.33	*SLC38A2, SLC6A6, SLC7A5, SLC7A11*
			Transmembrane	1.72	*ATP1B3, ELOVL6, ST8SIA5, BMPR2, EGFR, FZD10, GJA1, ITGA6, ITGAV, LAMP2, NRP1, SLC2A1, SLC38A2, SLC6A6, SLC7A5, SCD, TSPAN18, TFRC, TMEM41B, TYR*
			Lysosome	1.64	*LAPTM5, CTSD, ARL8A, LAMP2*
			Tube development	1.43	*SP3, NRP1, EPAS1, TP63, BMPR-II, BMPR1A*
			Enzyme linked receptor protein signaling pathway	1.33	*SMAD5, MADH2, BMPR1A, NRP1, BMPR-II, EGFR*
			Carboxylic acid biosynthetic process	1.22	*SCD, QKI, ELOVL6, CBS*
			Plasma membrane	1.15	*LAPTM5, BMPR1A, ITGAV, TJP1, SLC2A1, SLC6A6, BMPR-II, SLC38A2, LAMP2, ITGA6, QKI, EGFR, GJA1, PANX1, DSC1*
B	cEB vs. cEF	cEB	Translation	20.68	*RPL22, HARS, RPL35, RPL19, MRPL17, RPL10A, RPL27, RPL26, YARS, DENR, RPS14, RPL6, RPS4, RPS24, MRPL16, EF1A, RPL27A, RPL13, MRPL23, MRPS11, RPL4, RPS27A, EIF3J, RPL30, MRPL2, RPL18A, RPS8, RPL14, RPSA, RPL3, MRPL24, RPL37, RPS15, EEF1D, RPLP1, RPL8, RPS7, RPL31, RPS3, MRPS12, RPLP0, RPS28, COG8, RPL17*
			Oxidative phosphorylation	3.83	*NDUFA2, UQCR11, NDUFA8, ATP5A1W, NDUFB6, NDUFS5, COX4I1, NDUFAB1, COX6A1, COX15, ATP5H, UQCR10, NDUFB3, NDUFA1, NDUFB5, NDUFS6, COX5A, UQCRH, COX6C, ATP5B, ATP5G1, UQCRQ, COX7B, COX8A, ATP5O, NDUFA11*
			Ribosomal subunit	3.39	*RPL19, RPL26, RPS14, RPS27A, RPSA, RPS15, RPS3, DAP3, MRPS12, RPL17*
			Pyrimidine metabolism	2.07	*POLR2C, NME2, ITPA, POLR2L, POLR1D, POLR2H, POLR1C, CANT1, RPB6, POLR2I*
			Ubiquinol-cytochrome-c reductase activity	2.00	*UQCRH, UQCR10, UQCRQ*
			RNA polymerase	1.36	*POLR2H, RPB6, POLR1D, POLR2I, POLR1C, POLR2L, POLR2C*
			Transmembrance	2.76	*ATP1A1, ATP1B3, EPHB6, FAT, GPR177, ST8SIA5, ACVR1, CXCR7, EGFR, FGFR2, FGFR3, FZD10,GJA1, ITGA6, ITGAV, LAMP2, LAPTM4A, PTPLAD1, SLC22A5, SLC16A9, SLC2A1, SLC26A5, SLC39A13, SLC6A6, SLC7A5, SCD, SDC3, TSPAN18, TMEM121, TMEM175, TMEM41B, TYR, TYRP1*
		cEF	Tissue morphogenesis	1.59	*PRKAR1A, LMO4, TP63, TWSG1, JAG1, FGFR2, ACVR1, CA2*
			Regulation of ossification	1.58	*SMAD5, JAG1, FGFR2, HIF1A, ACVR1, WNT7B*
			Ossification	1.28	*SMAD5, TWSG1, FGFR2, MGP*
			Carboxylic acid transport	1.25	*SLC7A5, SLC6A6, SLC7A11, PLIN2*
			Glycoprotein metabolic process	1.18	*ST8SIA5, B3GNT5, B3GALNT1, HIF1A, B3GNT9*
			Transmembrane receptor protein serine/threonine kinase signaling pathway	1.13	*SMAD5, TWSG1, ACVR1, MADH2*
			Enzyme linked receptor protein signaling pathway	1.13	*EPHB6, JAK1, SMAD2, ACVR1, EGFR, FGFR2, FGFR3, RHOQ, SMAD5, TWSG1,*
C	cEF vs. cMF	cEF	Skeletal system development	2.81	*MGP, GLI1, WWOX, IGFBP5, GJA5, SHH, SOX14, SMAD1, CBFB*
			Signal peptide	2.29	*KITLG, NELL2, ADCYAP1, AGRN, APOA1, CTSD, CHRNA4, CRH, FMOD, FZD10, FRZB, IGFBP2, MGP, MXRA8, NFASC, NPY, PON2, PLTP, SFRP2, SEMA3A, SILV, SHH, TTR, TSKU, TYR, TYRP1, KIT*
			Tube development	1.62	*WNT5A, LMO4, LIPA, SHH, EDNRA, RARB, GJA5, CRH, GLI1*
			Melanin biosynthetic process	1.53	*TYR, TYRP1, PMEL*
			Drug metabolism	1.48	*GSTO1, GSTA, MGST2, ALDH3B1*
			Lytic vacuole	1.38	*CTSD, NAGA, CTSL2, SLC48A1*
			Developmental protein	1.38	*LFNG, BASP1, FZD10, FRZB, GLI1, MGP, MSX1, SFRP2, SEMA3A, SHH, TSKU, WNT2B, WNT5A*
			Regulation of transcription, DNA-dependent	1.26	*PKNOX2, POU2F3, SMAD1, SMAD2, SOX10, SOX14, AGRN, CBFB, DLX6, FOXI3, GLI1, HMGA2, MED22, MSX1, RHOQ, RBBP7, RARB, LOC425662, IRX5, SHH, THRB, TCEA2, TFAP2B, MYCL1, MYCN*
			Respiratory tube development	1.23	*WNT5A, LIPA, SHH, CRH, GLI1*
		cMF	Extracellular region	7.81	*HBEGF, COL3A1, STC2, CTGF, SS2, FN1, MMP2, ANXA2, QSOX1, SPARC, LAMC2, ST6GAL1, DKK3, ADIPOQ, ADM, COL6A1, GPC4, COL6A2, JSC, CD44, MDK, CYR61, SERPINI1, COL1A2, EREG, THBS2, LUM, IGFBP7, APOLD1, EPDR1, PLA2G12A, INHBA, LGALS1, TIMP3, FBLN1, LAMA3, COL8A1, ADAMTS1, CD109, NOV, COL4A1, COL4A2, OSF-2, AVD, ENSGALG00000016682, ENSGALG00000011930*
			Carbohydrate binding	2.99	*MRC2, HBEGF, CTGF, FN1, LAMC2, CD44, MDK, THBS2, CLEC3B, LGALS1, OSF-2*
			Signal peptide	2.62	*CD3E, GFRA4, K123, TIMP3, CDH5, COL1A2, COL3A1, COL6A1, COL6A2, COL8A1, CTGF, CYR61, DKK3, FBLN1, INHBA, ITGA6, LEPREL1, LUM, MMP2, LOC769899, MDK, PTGS2, QSOX1, SPARC, SERPINI1, SDK2, NOV, THBS2*
			Cell adhesion	2.25	*ITGB3, PPARD, CTGF, FN1, SDK2, CDH5, COL6A1, COL6A2, CD44, ITGA6, THBS2, EPDR1, COL8A1, EDIL3, OSF-2*
			Vasculature development	2.17	*CTGF, PRRX1, MMP2, ANXA2, CDH5, CYR61, CAV1, COL1A2, EPAS1, MYH9*
			Collagen	2.12	*ADIPOQ, COL1A2, COL3A1, COL4A1, COL6A1, COL6A2, COL8A1,*
			Phospholipid binding	1.50	*ANXA1, ANXA2, ANXA5*
			Vascular smooth muscle contraction	1.85	*ADRA1A, ITPR3, PLA2G4A, ACTG2, ARHGEF12, PLCB4, PLA2G12A, ITPR2, PLA2G10, RAMP2, PLA2G4C*
			Cell-substrate adhesion	1.40	*ITGB3, PPARD, CTGF, FN1, ITGA6, EPDR1*
			Regulation of cell growth	1.34	*CTGF, BCL6, CYR61, IGFBP7, INHBA, NOV*
			Cell surface	1.03	*ITGB3, HSPB1, II, CD3E, CD44, MDK, ITGA6*
D	cMF vs. cLF	cMF	Secondary metabolic process	2.33	*PMEL, TYRP1, ALDOB, TYR*
			Organic acid transport	2.16	*SLC6A6, CD36, PLIN2, OCA2*
			Signal peptide	1.53	*C1ORF187, FZD10, GSN, LY75, LOC769899, MDK, NPY, OVM, PLTP, QSOX1, SOSTDC1, SILV, SHH, TYR, TYRP1, KIT*
		cLF	Extracellular region part	5.09	*HBEGF, LAMB3, COL5A2, COL5A1, CTGF, FN1, SPARC, LAMC2, ADIPOQ, OSTN, LAMB1, THBS1, INHBA, LAMA3, COL12A1, COL4A1, OSF-2, MMP27, COL1A1, OVOS2*
			Structural molecule activity	3.43	*LOC395532, COL4A1, COL5A1, COL5A2, COL7A1, LOC395906, NEFL, RPL36, LOC771066, THBS1*
			Blood vessel development	2.47	*THY1, CDH2, CAV1, COL5A1, CTGF, THBS1*
			Glycoprotein	2.09	*ST6GALNAC2, ST6GAL1, THY1, ADORA1, ALPL, LOC395532, CDH2, CHST3, ENSGALG00000015908, FN1, HSP90B1, INHBA, LAMB1, NTM, P4HA2, SPARC, SERPINH1, MOXD1*
			Regulation of cell migration	1.98	*THY1, ADORA1, HBEGF, THBS1, LAMA3*
			Calcium binding	1.31	*ACTN1, CDH2, CALM1, SPARC, TNNC1*
			Glycoprotein biosynthetic process	1.31	*ST6GALNAC2, ST3GAL2, ST6GAL1, B3GNT2, GALNT1, CHST3*
			Cell adhesion	1.30	*THY1, NTM, COL5A1, COL7A1,CTGF, FN1, LAMB1, THBS1, CDH2, DSG2, EDIL3, POSTN*
			Negative regulation of molecular function	1.26	*THY1, ENSGALG00000014471, CAV1, HSPA5*
			Endoplasmic reticulum	1.22	*HSPA5, MOXD1, ITPR3, ADIPOQ, P4HA2, THY1, CAV1, SERPINH1, HSP90B1, DUOXA1*
			Identical protein binding	1.20	*TNNC1, HSPB1, ADIPOQ, INHBA, CAT, MTMR2*
			Regulation of cellular protein metabolic process	1.11	*ADIPOQ, PACSIN3, CAV1, THBS1NN BDKRB2*

The genes were analyzed by the Functional Annotation Clustering Tool. The top annotation clusters that had group enrichment scores greater than 1 were listed. The representative biology terms associated with the top annotation clusters are manually summarized

Genes involved in amino acid transmembrane transporter activity, lysosome, tube development, enzyme linked receptor protein signaling pathway, carboxylic acid biosynthetic process, and plasma membrane were increased significantly when the growth of a body feather turns to the plumulaceous portion. Six genes involved in tube development (*SP3*, *NRP1*, *EPAS1*, *TP63*, *BMPR-II*, *BMPR1A*) and six genes involved in enzymes linked receptor protein signaling pathway (*SMAD5*, *MADH2*, *BMPR1A*, *NRP1*, *BMPR-II*, *EGFR*) were up-regulated. Among these genes, TP63, a transcription factor of the p53 family, is known to be essential for the development of epidermis and its derivatives in vertebrates [33, 34]. *In situ* hybridization studies in chickens have shown that TP63 is highly expressed in the apical ectodermal ridge (AER) of the limb buds, interdigital tissues, epithelium of branchial arches, and feather buds [35]. Two receptors of BMPs were differentially expressed. Signaling via BMPRIA and BMPRIB is required to regulate intramembranous bone formation, chondrogenesis, and feather formation in chicken embryos [36]. The antagonistic balance between noggin and BMP4 has been shown to play a critical role in feather branching, with BMP4 promoting rachis formation and barb fusion, and noggin enhancing rachis and barb branching [19]. Epidermal growth factor (EGF) signaling is known to be required to pattern the feather array by promoting the interbud development [37].

### Transcriptomic comparison between pennaceous body and flight feathers

Among the 1,287 DEGs between pennaceous body and flight feathers, 988 were up-regulated and 299 genes were down-regulated in the pennaceous body feather (Fig. 4b, Additional file 7: Table S6). IPA canonical pathway analysis showed that these DEGs included several genes involved in the Sertoli cell-Sertoli cell junction signaling (*PVRL1*, *TJP1*, *TUBB3*, *CLDN3*, *CLDN4*, *CGN*, *TUBB4B*, *RAB8B*, *PRKAR1A*, *MAP3K1*, *TUBA1B*, *MAPK1*, *TUBA4A*, *TJP3*) and in the regulation of the epithelial-mesenchymal transition pathway (*FGFR2*, *FGFR3*, *EGFR*, *PDGFD*, *PARD6A*, *JAG1*, *CLDN3*, *SMAD2*, *FZD2*, *JAK1*, *PYGO2*, *HIF1A*, *MAPK1*, *WNT6*, *HMGA2*) (Fig. 5, Additional file 11: Table S10).

Among the up-regulated genes in the pennaceous portion of the body feather, the expression of genes involved in translation, oxidative phosphorylation, pyrimidine metabolism, ribosomal subunit, ubiquinol-cytochrome-c reductase activity, and RNA polymerase was increased significantly (Table 2). The up-regulation of genes in these groups may be due to the need of large quantities of protein synthesis in the pennaceous portion of the body feather.

Genes involved in skeletal system development, tube development, melanin biosynthetic process, regulation of RNA metabolic process, drug metabolism, respiratory tube development, and lytic vacuole were expressed significantly higher in early-grow flight feather (Table 2). The genes in melanin biosynthetic process (*TYR*, *TYRP1*, *PMEL*) were up-regulated simply because the color is usually darker in the flight feather of this breed than in the contour feather of both TCC_L2 and white leghorn chickens. Tyrosinase (TYR) and tyrosinase-related protein 1 (TYRP1) are known to be involved in the feather pigment pattern formation [38]. TYR and TYRP1 are found to be associated with melanic plumage color differences in chickens, Korean quails (Coturnix coturnix), ducks, geese, and pigeons [39–43]. The mutation of premelanosome protein (PEML) can cause hypopigmentation in chickens [44].

Several genes involved in tissue morphogenesis (*PRKAR1A*, *LMO4*, *TP63*, *TWSG1*, *JAG1*, *FGFR2*, *ACVR1*, *CA2*) and in the regulation of developmental process (*SMAD5*, *JAG1*, *FGFR2*, *HIF1A*, *ACVR1*, *WNT7B*) were upregulated in distal flight feather (Table 2). Jagged-1 (JAG1), a Notch ligand, is involved in the orientation of feather bud elongation [45]. Three fibroblast growth factor (FGF) receptor genes, FGFR1, FGFR2 and FGFR3, have been suggested to be involved in feather morphogenesis [46].

### Transcriptomic comparison between distal and proximal parts of flight feather

Toward the proximal end, the rachis gradually increases in width and eventually turns into the calamus. Among the 1,167 DEGs, 534 genes were up-regulated and 633 genes were down-regulated in the proximal flight feather in comparison to the distal flight feather (Fig. 4c, Additional file 8: Table S7). IPA canonical pathway analysis revealed several DEGs that were involved in WNT/β-catenin Signaling (*JUN*, *DKK3*, *WNT5A*, *CDH3*, *CD44*, *CDH5*, *PPARD*, *WNT2B*, *SFRP4*, *SOX14*, *SFRP2*, *PPP2R2B*, *SOX7*, *FRZB*, *RARB*) and in TGF-β Signaling (*JUN*, *INHBA*, *SMAD2*, *SMAD1*, *RUNX3*, *MAPK11*, *INHBB*) (Fig. 5, Additional file 12: Table S11). Other pathways basically overlap with the WNT/β-catenin signaling or the TGF-β signaling pathway.

Nine genes involved in skeletal system development (*MGP*, *GLI1*, *WWOX*, *IGFBP5*, *GJA5*, *SHH*, *SOX14*, *SMAD1*, *CBFB*) were increased in expression in the early-grow (distal) flight feather (Table 2). BMP4 and matrix gla protein (MGP) are considered an activating and an inhibitory morphogen, respectively, and their interaction is important for vascular branching [47]. MGP may promote rachis and barb branching in feather. SHH is a secreted protein expressed in the epidermis that is involved in the mitogenic and morphogenetic processes throughout feather development [32, 48–52]. The interactions between SHH and BMP2 signaling during feather barb ridge morphogenesis may be critical for the initial formation of a meristic pattern of barb ridges and the variation in barb morphogenesis in feathers [53]. The activation of the SHH signaling pathway leads to the expression of the transcription factor glioma-associated oncogene 1 (GLI1), a SHH-targeted mediator [54]. Insulin-like growth factor binding protein 5 (IGFBP5) is expressed in human hair follicle dermal papilla and plays a specific role in the local modulation of IGF action during the hair growth cycle [55].

Nine genes involved in tube development (*WNT5A, LMO4, LIPA, SHH, EDNRA, RARB, GJA5, CRH, GLI1*) were increased in expression in the early-growth flight feather (Table 2). The expression levels of WNT ligands such as WNT5A/WNT5B/WNT6 were found to be high in the feather epithelium and pulp compared to dermal papillae [56]. WNT5A is involved in non-canonical pathways but its downstream signaling events are not known yet. LIM domain-only protein 4 (LMO4) is expressed in mouse hair follicles, especially in the sebaceous glands, undifferentiated bulb cells, and the outer epithelial root sheath [57]. Retinoic acid receptor beta (RARB) is a receptor of retinoic acid which regulates cell proliferation, differentiation, and morphogenesis and is involved in the feather-bud formation [58]. Gap junction alpha-5 protein (GJA5), also known as connexin 40 (CX40), is an integral membrane protein that oligomerizes to form intercellular channels that are clustered at gap junctions which are present in supportive cells located in the vicinity of barbule cells [59]. Corticotropin-releasing hormone (CRH) peptides modulate human hair growth/cycling [60, 61].

Many genes involved in extracellular region and cell adhesion were up-regulated significantly in the middle-grow flight feather (Table 2). Cell adhesion molecules (CAMs) may regulate feather morphogenesis by constraining cell motion and forming borders. Several adhesion molecules, including L-CAM, N-CAM, integrin, tenascin, as well as proteoglycan, are involved in feather development [62–64]. Tenascin-C has been shown to evolve rapidly in avian lineages [29].

Many collagen genes were up-regulated in the middle-grow feather portion compared to the early-growth flight feather portion (Table 2). The orientation of collagen fibers in the feather buds may promote feather growth by creating a gradient of stiffness, thus triggering the pressure sensitive growth factors [65]. Collagen types I and III, and fibronectin are known to be involved in feather morphogenesis in the chick embryo [66]. Matrix metalloproteinases (MMPs) and their inhibitors are important in tissue development remodeling for the formation of feather follicles such as epithelium invagination and mesenchymal cell proliferation [67]. Several collagens and a MMP expressed in feathers have been found to evolve rapidly in a previous study [29]. Dickkopf-related protein 2 (DKK2), which presumably encodes a WNT signaling inhibitor, regulates feather regeneration in the dermal papillae [56]. The expression of CD44, which is also known to evolve rapidly in avian lineages [29], correlates with the onset of keratinocyte stratification and mesenchymal maturation into fibrous dermis in fetal human skin [68]. Tissue inhibitor of metalloproteinases-3 (TIMP3) is expressed in epithelial outer root sheath cells of growing hair follicles of human fetus [69]. Cysteine-rich secretory protein 1 (CRISP1) is expressed in murine hair follicles and down-regulated in mice overexpressing a homeobox gene HOXC13 [70].

State-dependent signaling by Cav1.2 regulates hair follicle stem cell function by regulating the production of the bulge-derived BMP inhibitor follistatin-like1 (FSTL1), derepressing stem cell quiescence [71, 72]. Expression of muscle-related genes are known to be enriched in the feather dermal papilla, including *ACTG2* (smooth muscle actin, gamma 2), *ACTA2* (smooth muscle actin, alpha 2), Desmin, MYH11 (myosin heavy chain11), *MYL4* (myosin light chain4), *MYL9* (myosin light chain 9), *MYLK* (myosin light chain kinase), etc. [56]. Our results showed that genes involved in smooth muscle contraction, such as *ADRA1A*, *ITPR3*, *PLA2G4A*, *ACTG2*, *ARHGEF12*, *PLCB4*, *PLA2G12A*, *ITPR2*, *PLA2G10*, *RAMP2*, and *PLA2G4C* are differentially expressed. CLR/RAMP2-overexpressing mice revealed a defined phenotype with thinning of the hair during postnatal development [73].

### Transcriptomic comparison between proximal flight feather and calamus

Among the 702 DEGs, 263 genes were up-regulated and 404 genes were down-regulated in the proximal flight feather in comparison to the calamus (Fig. 4d, Additional file 9: Table S8). IPA canonical pathway analysis showed that several genes involved in the TGF-β signaling (*INHBA, RUNX3, PMEPA1, RUNX2, INHBB*), the Sertoli cell-Sertoli cell junction signaling (*TUBB3, TUBA1B, CLDN4, TJP3, JAM3, ACTN1, MTMR2*) and the germ cell-Sertoli cell junction (*CDH2, TUBB3, GSN, TUBA1B, ACTN1, MTMR2*) signaling were differentially expressed. Other pathways basically overlap with the TGF-β signaling pathway (Fig. 5, Additional file 13: Table S12).

Compared to the close proximal part of the flight feather, the calamus expressed significantly more genes involved in extracellular matrix and cell adhesion (Table 2). The calamus can basically be seen as the rachis of the flight feather. The genes involved in extracellular matrix and cell adhesion may be required for making a tougher feather structure.

### The molecular mechanism of feather branching morphogenesis

Major signaling pathways are involved in feather branching morphogenesis, including the Wnt/β-catenin, SHH/BMP, and Notch pathways [11, 18, 19, 45, 48, 56, 74, 75]. Besides feathers, epithelial tissues such as the vascular system, kidney, lung, and mammary gland arise through branching morphogenesis of a pre-existing epithelial structure [13, 76–78]. Common morphological stages and a similar set of developmental regulations are shared by these tissues. The spatial and temporal controls of branching are controlled by developmental decisions requiring regulation of cell proliferation, apoptosis, invasiveness, and cell motility. Similar molecular mechanisms could exist for the epithelial branching program, even though the feather is an evolutionary novel tissue. Key branching morphogenetic molecules include central signaling molecules such as BMPs, TGF-β, FGF, and MMPs [13, 76–78]. Our study supports the previous findings that temporal and spatial variation of BMP signals are critical for generating branching differences between pennaceous and plumulaceous body feathers because genes involved in BMP signaling were significantly upregulated in the plumulaceous portions.

Genes involved in axon guidance (*MYL4, CDK5, SEMA4B, PRKAR1A, NFATC3, PLXNA1, PLXNB2, ARPC4, NRP1, MAPK1, WNT6, PRKCI, RASA1, ECE2, SEMA5A*) from IPA pathway analysis (Fig. 5), especially those in semaphorin signaling, were differentially expressed between the pennaceous and plumulaceous portions of body feather. This observation suggests that they are recruited in feather development and play a critical role in controlling the morphological differences in feathers, and perhaps are involved in changing the extracellular environment for signals that instruct the cell of the barb plate which direction to grow by affecting the cytoskeleton. The differential expression profile of these genes among different feather types suggests that they are involved in critical guidance cues during feather morphogenesis, although functional studies remain to be demonstrated.

Genes involved in Sertoli cell-Sertoli cell junction signaling and germ cell-Sertoli cell junction are recruited in feather development. Differentiating barb/barbule cells have been found to have many adhesion junctions, some gap junctions and fewer tight junctions during early stages of feather development [59]. The cytological details on the type of cell junctions present in barb/barbules of feathers are poorly known. Our study provides the data to characterize the types of cell junctions, and their molecular nature that are critical in feather morphogenesis.

Although the hair and the feather are not homologous, they share many pathways. Hair follicle morphogenesis also depends on WNT, SHH, NOTCH, BMP and other signaling pathways that interact between epithelial and mesenchymal cells. However, as hairs have no branching structures, the genes that are involved in feather morphogenesis but not in hair development may participate in generating branching structures. A detailed comparison of transcriptomes between feathers and hairs may reveal the molecular mechanism shared and distinct between these two types of keratinized skin appendages. The genes involved in vessel and tube development are differentially expressed in feather epithelium, suggesting a role in regulating the morphology of feather branching. We found that genes involved in developing the vessel, tube and kidney were enriched. They may have been co-opted to develop an evolutionary novelty. The origin and diversification of a novel structure may not require the evolution of new gene or gene duplication because existing genes can be recruited to have new expression pattern and regulation. The genes with similar functions can be reused to construct a new network.

## Conclusions

This study has significantly increased our understanding of the expression profiles of feather related genes. We examined the expression profiles of genes associated with the development of feather structure and compared the gene expression patterns in different types of feathers and different portions of a feather to advance our understanding of the molecular mechanisms of feather growth and the molecular basis of variation in feather structure. Our results are a valuable resource for understanding the molecular mechanisms of avian feather development. This study produced abundant data for the analysis of gene expression during feather morphogenesis. Morphotype-specifically expressed genes were identified from five zones of feather filament epithelia. Some identified genes may be associated with the growth control during feather regeneration, the formation of special branching structures, or barb differentiation themselves. The present study provides a basis for future study of the complex molecular and cellular events during feather development.

## Methods

### Animals

All the animals used in this study were processed following the approved protocol of Institutional Animal Care and Use Committees of the National Chung Hsing University (Taichung, Taiwan). For total RNA extraction, we used the Taiwan County Chicken (TCC_L2) breed chicken for wing flight feathers and white leghorn for body contour feathers. TCC_L2 and white leghorn chicken contour feathers are different in color but highly similar in morphology and structure.

### Total RNA Isolation and RNA-seq

We collected regenerating pennaceous and plumulaceous portions of body contour feathers, the distal and proximal portions of primary flight feathers, and the calamus of primary flight feathers. Total RNA was isolated from early or late grow fresh feather epithelial tissues corresponding, respectively, to the distal and proximal part of a feather (Additional file 1: Figure S3), which was dissected from the follicle tissue and separated from the mesenchyme in Calcium-Magnesium Free Saline (CMFS 2X) on ice [79]. White leghorn chickens were used for body contour feathers to avoid melanin contamination, which is difficult to remove and can inhibit essential enzymatic reactions for RNA-seq [80, 81]. RNA-seq and analysis of paired-end reads were performed as described in Ng et al. 2014 [28]. Reads were mapped onto the chicken genome assembly ICGSC Gallus_gallus-4.0 (GCA_000002315.2).

### Validation by real-time quantitative PCR

A total of 2 μg RNA of each sample was reverse transcribed with MultiScribe Reverse Transcriptase (Thermo Fisher Scientific, Waltham, MA) into cDNA for both Reverse Transcription PCR (RT-PCR) and Quantitative Reverse Transcription PCR (qRT-PCR) reactions. Total RNA was incubated with RT enzymes at 25 °C for 10 min prior to the RT reaction. RT reactions were performed at 37 °C for 2 h followed by the inactivation of RT enzyme at 85 °C for 10 s. For RT-PCR, 1 μl of 10× diluted cDNA was amplified by Fast Start *Taq* DNA polymerase (Roche Applied Science, Penzberg, Germany) in a total of 10 ul reaction. For RT-qPCR, 1 μl of 10× diluted cDNA products was quantified with 2 × SYBR Green Master Mix (Kapa Biosystems, Wilmington, MA) in a total of 10 ul reaction and performed on a Roche LightCycler 480 Instrument II. All the data were analyzed by the HTC1 software (Roche Applied Science). The 2^-ΔΔCt^ method was used to calculate relative expression levels [82]. The cycling parameters of RT-qPCR were as follows: 95 °C for 3 min, then 40 cycles of 95 °C for 10 s, and annealing for 20 s. Gene names and primer sequences are shown in Additional file 3: Table S2. Each sample was analyzed in duplicates, and gene expression levels were normalized against the corresponding TATA-binding protein (*TBP*) expression level.

### Multivariate analyses

Prior to statistical analyses performed with R v2.15.3 (R Development Core Team 2011), raw read counts were normalized by Fragments Per Kilobase of transcript per Million mapped reads (FPKM). Principal component analysis (PCA) was performed on the covariance matrix f using a custom R script based on the “prcomp” R package.

### Identification of differentially expressed genes

We used the non-parametric method to identify differentially expressed genes (DEGs) between two samples [83]. Here, we set the *q* value (differentially expression probability) in the method to be 0.75 (this value is equivalent to an odd of 3:1, i.e., the gene is three times more likely to be differentially expressed than it is not) and require at least a 2-fold change in FPKM between the two samples.

### Gene functional annotation, canonical pathway and upstream regulator analyses

Functional annotations of gene loci were compared with the complete genome using annotations from the Database for Annotation, Visualization, and Discovery (DAVID), which uses fuzzy clustering to group genes into functionally related classes based on the similarity of their annotations [84, 85]. Pathway analyses of differentially-expressed genes were carried out using the Ingenuity Pathways Analysis software (IPA; Ingenuity Systems, www.ingenuity.com). Each gene identifier was mapped to its corresponding gene object in the Ingenuity Pathways Knowledge Base. A canonical pathways analysis was generated to identify the pathways from the IPA library that were most significant. Fischer’s exact test was employed to calculate the *p*-value which determines the probability that each biological function or/and canonical pathway is due to chance alone. The Upstream Analysis section of the core analysis was used to determine which upstream regulators were associated with the observed differently expressed genes.

### Availability of supporting data

The full data sets have been submitted to NCBI Sequence Read Archive (SRA) under accession nos. SRX528281, SRX528834, SRX528843, SRX529337, SRX529339, SRX529353-SRX529362. Bioproject: PRJNA245063.

## Additional files

Additional file 1: Figure S1. The feather samples used in this study. **Figure S2.** qPCR validation of 10 genes with biological replicates. **Figure S3.** The feather samples used for RNA extraction. (PDF 270 kb) Additional file 2: Table S1. Summary of the 15 feather epithelial transcriptomes. (XLSX 15 kb) Additional file 3: Table S2. Gene name and primer sequences used in RNA-seq validation. (XLSX 12 kb) Additional file 4: Table S3. Transcriptome Expression Data. Table of mapped reads to Galgal4 transcripts for all 15 data sets. FPKM (Fragments per kilobase of exon per million fragments mapped): normalized transcript abundance values for each gene in the indicated tissues. (CSV 1314 kb) Additional file 5: Table S4. Positively selected and/or rapid evolving genes in avian lineages [29] that are expressed in all feather samples. (XLSX 11 kb) Additional file 6: Table S5. cEB vs. cLB differentially expressed gene set (shown in FPKM and fold change). (XLSX 81 kb) Additional file 7: Table S6. cEB vs. cEF differentially expressed gene set (shown in FPKM and fold change). (XLSX 94 kb) Additional file 8: Table S7. cEF vs. cMF differentially expressed gene set (shown in FPKM and fold change). (XLSX 86 kb) Additional file 9: Table S8. cMF vs. cLF differentially expressed gene set (shown in FPKM and fold change). (XLSX 62 kb) Additional file 10: Table S9. Canonical pathways for cEB vs. cLB differentially expressed gene set. (XLS 50 kb) Additional file 11: Table S10. Canonical pathways for cEB vs. cEF differentially expressed gene set. (XLS 35 kb) Additional file 12: Table S11. Canonical pathways for cEF vs. cMF differentially expressed gene set. (XLS 46 kb) Additional file 13: Table S12. Canonical pathways for cMF vs. cLF differentially expressed gene set. (XLS 30 kb)

## Abbreviations

FPKM: Fragments Per Kilobase of transcript per Million mapped reads
RT-qPCR: Real time quantitative PCR
DEGs: Differentially expressed genes
PCA: Principal components analysis
GO: Gene ontology
DAVID: Database for Annotation, Visualization, and Discovery
IPA: Ingenuity pathways analysis
