# Supplementary material for: Transcriptomic analyses of regenerating adult feathers in chicken
Source: BMC Genomics. 2015 Oct 6;16:756. doi: 10.1186/s12864-015-1966-6 (PMC4594745; doi:10.1186/s12864-015-1966-6)
Supplement: Additional file 1: Figure S1. — The feather samples used in this study. Figure S2. qPCR validation of 10 genes with biological replicates. Figure S3. The feather samples used for RNA extraction. (PDF 270 kb) [file 12864_2015_1966_MOESM1_ESM.pdf]

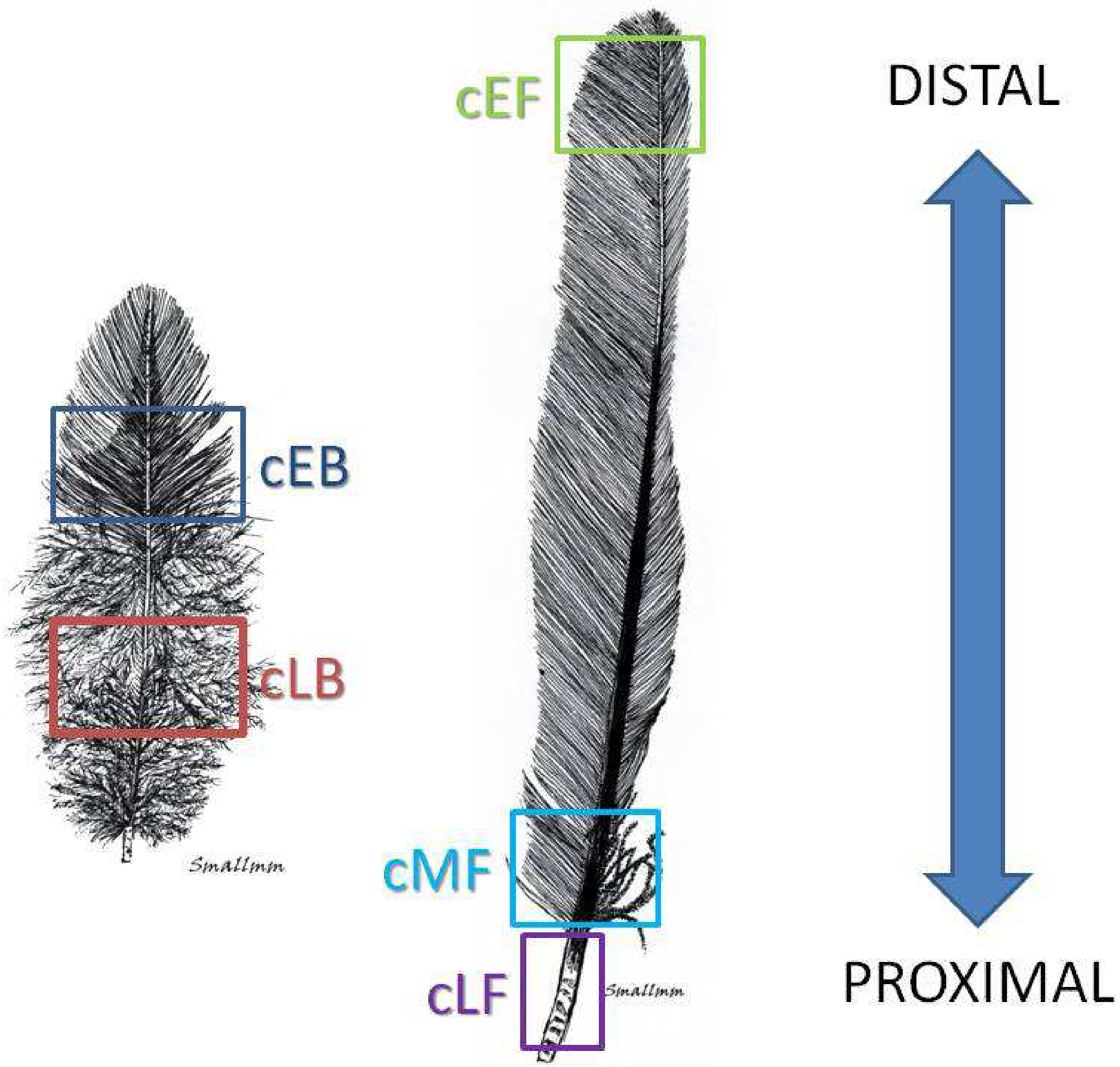

Figure S1

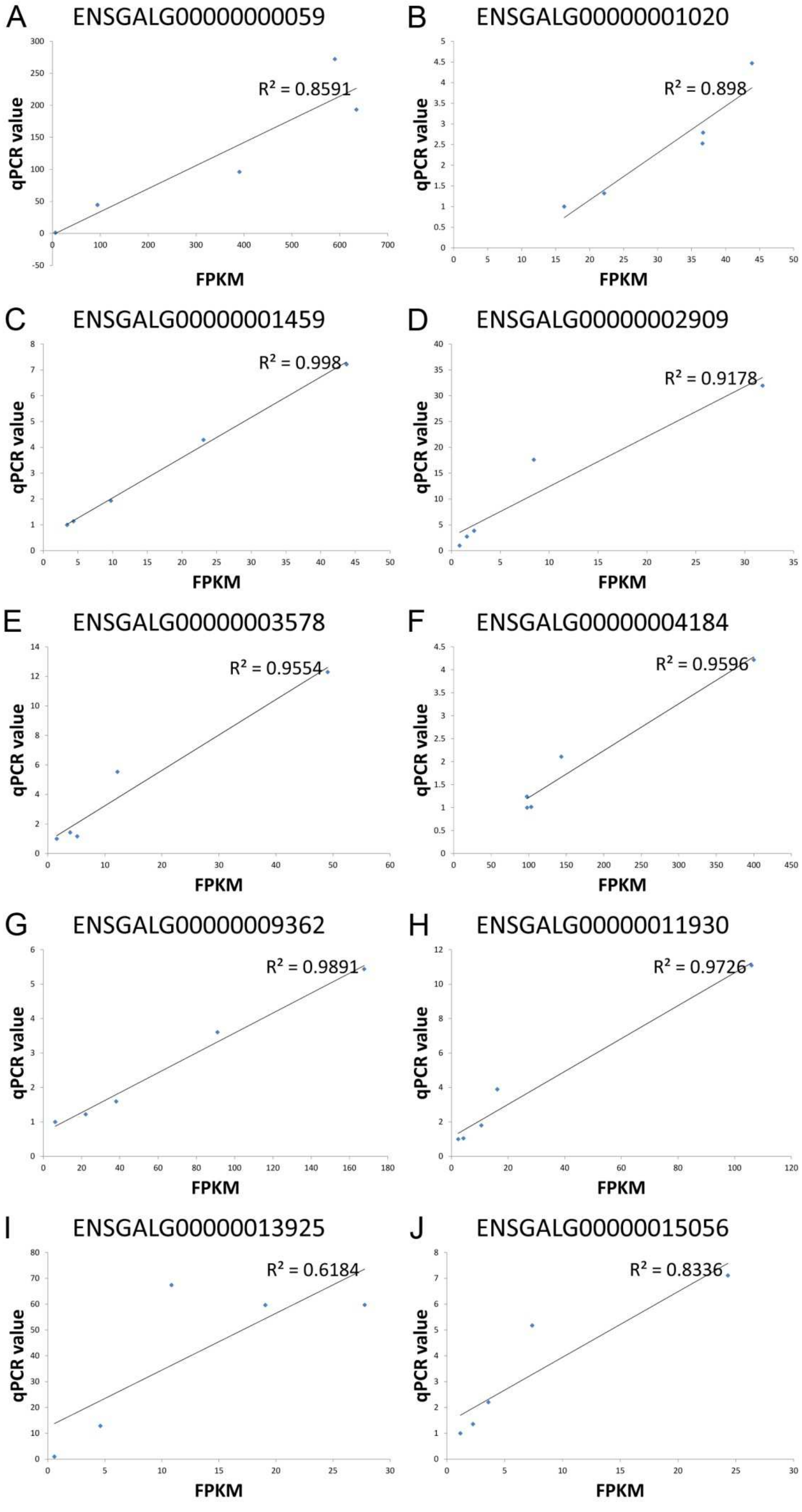
A ENSGALG00000000059
R² = 0.8591
B ENSGALG00000001020
R² = 0.898
C ENSGALG00000001459
R² = 0.998
D ENSGALG00000002909
R² = 0.9178
E ENSGALG00000003578
R² = 0.9554
F ENSGALG00000004184
R² = 0.9596
G ENSGALG00000009362
R² = 0.9891
H ENSGALG00000011930
R² = 0.9726
I ENSGALG00000013925
R² = 0.6184
J ENSGALG00000015056
R² = 0.8336
qPCR value
FPKM

Figure S2

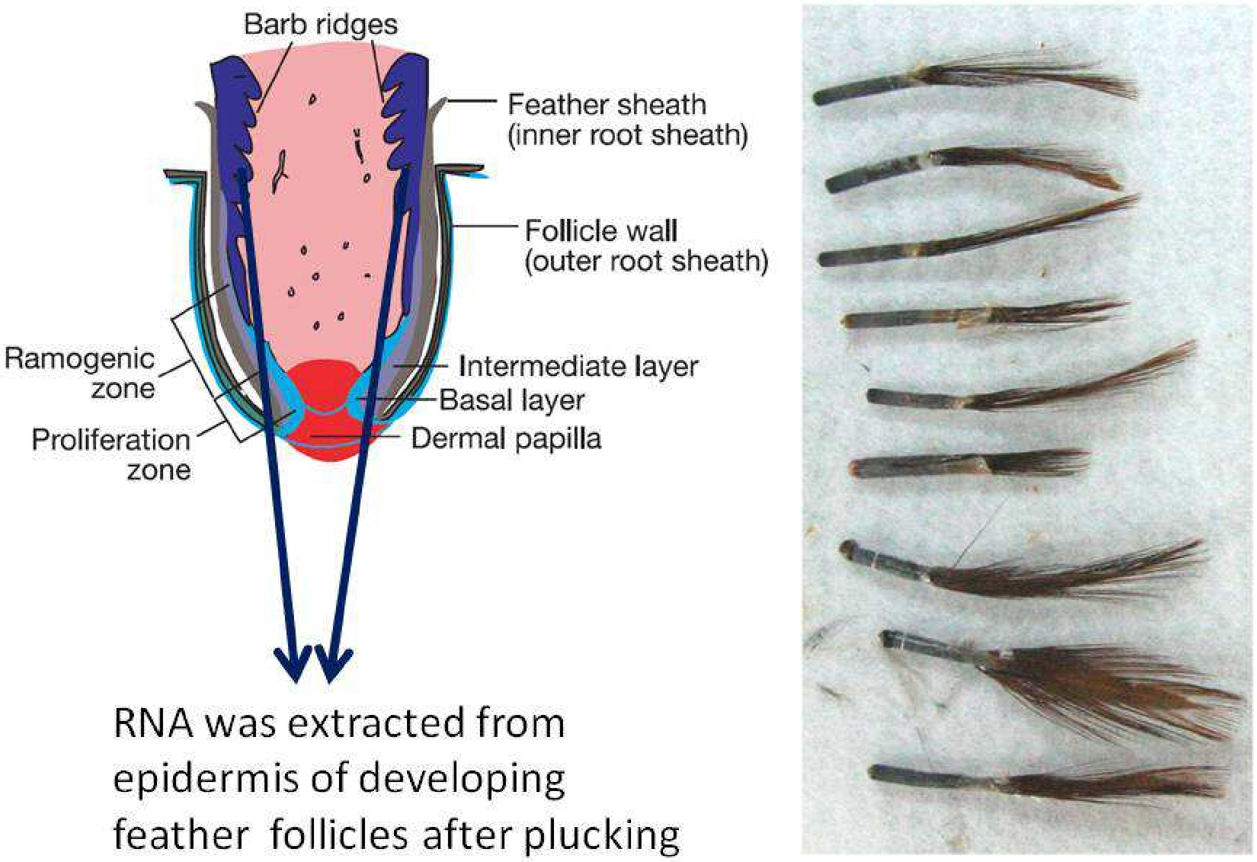
Barb ridges
Feather sheath
(inner root sheath)
Follicle wall
(outer root sheath)
Ramogenic
zone
Intermediate layer
Basal layer
Proliferation
zone
Dermal papilla
RNA was extracted from
epidermis of developing
feather follicles after plucking

Figure S3
